# Contribution to the Detection of Poor Quality Sildenafil Drugs in Burkina Faso Using High-Performance Thin-Layer Chromatography

**DOI:** 10.1155/2021/4093859

**Published:** 2021-10-13

**Authors:** Abdoul Karim Sakira, Mitéhélé Sandrine Marie Josiane Ouattara, Moussa Yabre, Moumouni Bande, Touridomon Issa Some

**Affiliations:** Laboratoire de Toxicologie, Environnement et Santé (LATES), Ecole Doctorale des Sciences de La Santé (ED2S), Université Joseph KI-ZERBO, 03 BP 7021 03, Ouagadougou, Burkina Faso

## Abstract

In substandard drugs enforcement, there is a need to develop reliable, fast, and inexpensive analytical methods. Due to its very characteristics, HPTLC offers opportunities for the development of methods that meet these requirements. This technique was used to develop and validate a method for the determination of sildenafil in pharmaceutical formulations from the licit and illicit supply chain in Burkina Faso. Taking into account optimization parameters such as measurement wavelength and mobile phase composition, the best elution quality is found at the maximum signals of spots on silica plates at 305 nm, using a mixture of dichloromethane-methanol mixture 9 : 1 (v/v) proportions. The method developed under these conditions was validated using the accuracy profile as a decision tool. The establishment of the response function curves allowed the choice of the polynomial function applied to the peak areas. This mathematical model provides a validity range between 0.4 and 0.6 mg/mL. The application of the developed and validated method to collected samples allowed the detection of two substandard drugs and confirmed the poor quality of drugs in the illicit market. More data using this approach in a variety of drug molecules could lead to the establishment of databases of counterfeit drugs in Burkina Faso.

## 1. Introduction

Data from the literature show that the market share of illicit or falsified drugs is estimated at 10% with an increasing trend in some countries. All therapeutic classes are concerned. These falsified drugs cover a range from products with unknown sources to those with or without active ingredient, under- or overdosed, or with falsified expiration date [1–5].

Falsified or poor quality medicines are a serious concern to public health worldwide, especially for developing countries where surveillance systems are weak and quality control of drugs is inefficient. The quality control of medicines is generally based on the monographs of the different pharmacopoeias and particularly from WHO. Counterfeiting analysis can be done by simple visual inspection or through the use of sophisticated techniques including imaging, spectrometric, and chromatographic techniques. The analytical methods to be used are classified into two main groups: destructive methods and nondestructive methods; the latter can be performed outside the laboratory. Among the destructive methods are nuclear magnetic resonance (NMR), atomic absorption spectrophotometry, gas chromatography, and liquid chromatography coupled with diode array or mass spectrometer detector and thin-layer chromatography [2, 6]. Nondestructive methods include X-ray diffraction, mid- and near-infrared spectrophotometry, and Raman spectrophotometry.

In drug counterfeiting enforcement, it is necessary to have simple, fast, and efficient methods for the analysis of suspect products, and High-Performance Thin-Layer Chromatography (HPTLC), by its very characteristics, offers an opportunity to achieve these objectives. This technique uses a simple and reliable apparatus, which does not require large quantities of solvents, is not time-consuming compared to HPLC, and minimizes the effect of the matrix on the analysis. Automation of deposition and quantification and improved quality of stationary phases combined with ready-to-use analytical plates make HPTLC robust, fast, and sensitive which can be coupled with satisfactory sensitivity detectors such as the photodensitometer and mass detector [7–9]. As a result, HPTLC is gradually gaining acceptance in regulatory monographs and in particular for the analysis of natural extracts where matrices are often very complex [7, 10, 11]. In addition, due to its relatively affordable cost per analysis, it appears to be an excellent alternative adapted to the context of developing countries, to control the quality of various products, particularly pharmaceuticals. HPTLC has already been successfully implemented in these countries for the quality control of various pharmaceutical products such as antituberculosis, antibiotics, antifungals, and antiretroviral drugs [12, 13]. In this study, we developed and validated a method for the analysis of pharmaceutical formulations of sildenafil from the licit and illicit distribution chain, using HPTLC. This molecule is a phosphodiesterase type 5 inhibitor, used in erectile dysfunction, which since its introduction has been frequently used in adulterations of herbal products and dietary supplements [14–16]. This study will contribute to generating data on the issue of counterfeiting in Burkina Faso.

## 2. Materials and Methods

### 2.1. Sampling

All the drugs analyzed were collected in the Central, Central-East, South-West, and South-Central regions of Burkina Faso. Samples from the licit supply chain were purchased in pharmacies and private pharmaceutical stores. Samples from the illicit market came from different places in the same regions. The characteristics of the samples collected are noted in Table 1.

### 2.2. Reagents and Standards

Reagents and analytical standards used for the implementation of the HPTLC method were as follows: methanol (HiPerSolv purity ≥ 99,8%), dichloromethane (HiPerSolv purity ≥ 99,8%), chloroform (HiPerSolv CHROMANORM, purity ≥ 99,8%), acetone (HiPerSolv CHROMANORM) all from VWR; ammonium hydroxide solution (25%) from Honeywell Fluka™); and diethylamine (purity ≥ 99.5%) from Sigma-Aldrich.

Sildenafil citrate (purity ≥ 98%) was from Sigma-Aldrich and Viagra® tablets 50 mg B/4 B226423B from Pfizer limited. ERECTAD® (tadalafil tablets) 20 mg B/4 AT2E-1601 was from ASMOH Laboratories Limited.

### 2.3. Instrumentation

A High-Performance Thin-Layer Chromatography line comprising of several modules was used. Automatic TLC Sampler 4 (CAMAG, Switzerland) was used for the application of the samples. Chromatographic development was done using a Twin trough chamber (20 × 10 cm; 20 × 20 cm Camag, Switzerland). An UV chamber (Camag, Switzerland) made it possible to visualize the chromatographic plates (Merck TLC silica gel F_254_20 × 10 cm). The detection and the quantitative analysis of the strips were carried out using the photodensitometer TLC scanner 4 (Camag, Switzerland). The operating parameters have been set using the vision CATS® 2.4 software.

### 2.4. Preparation of Calibration and Validation Standard Solutions

A stock solution of sildenafil citrate at 1.0 mg/mL was prepared by dissolving exactly 20 mg of powder in 20 mL of methanol. The mixture was stirred in an ultrasonic bath (sonicator) for 20 minutes. Aliquots of the stock solutions were appropriately diluted with methanol to obtain working standards of 0.1 mg/mL, 0.3 mg/mL, and 0.7 mg/mL. The Viagra® sample used for the validation of the method on the matrix was prepared under the same conditions. It was diluted to the same concentrations as the daughter solutions of the standard's calibration, that is, approximately 0.1 mg/mL, 0.3 mg/mL, and 0.7 mg/mL.

### 2.5. Sample Solutions Preparation

Depending on the packaging, at least three tablets per sample from the same batch were weighed and then crushed. A quantity of powder equivalent to approximately 10 mg of sildenafil citrate was weighed and placed in a 10 mL volumetric flask. Approximately, 7 mL of methanol was added and the solution was vortexed for 5 minutes and then 20 min in the ultrasonic bath. The volume was completed with methanol just after. The concentration of the sample stock solution of sildenafil citrate was 1 mg/mL. The resulting solution was filtered through a 0.45 *μ*m PTFE filter, and then 5 mL of the filtered solution was transferred in a 10 mL volumetric flask. The final concentration of the sample solution was 0.5 mg/mL of sildenafil. The obtained working solutions were conditioned in 1.5 mL vials.

### 2.6. Chromatographic Procedure: Optimization

The influence of several operational and instrumental parameters during the optimization of the method depending on the substance and the matrix can be analyzed in HPTLC. In the present study, the vertical mode plate development and the deposition of samples in strip form were retained. The influence of mobile phase composition and wavelength were tested. Three different mobile phases and six wavelengths used in previous HPTLC sildenafil assay studies were tested [17–19]. For the mobile phases, the following systems were used:

- Mobile phase 1: dichloromethane-acetone-hydroxide ammonium (20 : 1.5: 0.2, v/v/v) and (10 : 10: 0.2, v/v/v)
- Mobile phase 2: chloroform-diethylamine-methanol (15 : 3: 2, v/v/v)
- Mobile phase 3: dichloromethane-methanol (9 : 1, v/v)

A wavelength range from 254 nm to 366 nm was tested and the wavelengths, namely, 254 nm, 278 nm, 292 nm, 305 nm, 312 nm, and 333 nm, were selected after optimization. The final selected operating conditions are summarized in Table 2.

### 2.7. Identification and Sildenafil Quantification

Densitometric scanning was performed using a Camag TLC Scanner 4, and the densitograms are generated by the visionCATS® 2.4 software. The strip or spot readings are made at different wavelengths in absorption-reflection mode. The following scanning parameters were used: slit dimension (5.00 × 0.20 mm, micro), scanning speed 20 mm/s, and data resolution 100 *μ*m/step. The scanning was done between 190 and 900 nm range using deuterium and tungsten lamps.

The quantification is based on peak areas peak height. The concentrations are calculated based on the polynomial regression model using the standard concentrations versus reflected intensities. The identification of sildenafil is made by comparing the frontal reference (ratio) of the sildenafil standard and that of the analyzed sample. This frontal ratio corresponds to the migration distance of the substance over the solvent front.

### 2.8. Method Validation

The validation strategy is the accuracy profile, a graphical and visual decision tool for the validation of assay methods [20–22]. This validation strategy combines two fundamental elements of validation, namely, the accuracy and precision of the final measurement result. It, therefore, takes into account the total measurement error (systematic + random). Accuracy profile validation is based on the estimation of a range within which the result of a measurement lies and the verification that it is within the set limits. The principle is based on the statement that the difference between a measure (*x*) and its true value (*μ*) must be less than the predefined acceptance limit (*λ*): −*λ* < *x* − *μ* < *λ*⟺|*x* − *μ*| < *λ*.

#### 2.8.1. Validation Criteria Used

The validation plan applied is summarized in Table 3 [23].

*Specificity of the Method*. The evaluation of this parameter was based on the discrimination of the sildenafil peak with those of other present or chemically similar substances such as tadalafil. This discrimination takes into account the evaluation of the purity of the peaks through their forms [24]. It was estimated by calculating the resolution parameter that takes into account the frontal ratios recorded on the chromatographic profiles.

*Tolerance Interval and Accuracy*. The proposed method would be validated if the different tolerance limits of the results remain within the acceptance limits in a given interval called the validity interval. The probability ß associated with the tolerance interval is 80% and the acceptance limits are ±10% [25]. The accuracy profile was constructed based on the trueness and precision data.

*Trueness*. It is expressed in terms of bias (mg/ml), relative bias (%), and recovery.

*Precision*. The precision was analyzed in terms of repeatability and intermediate precision. To provide an indication of errors due to chance, the precision was expressed in terms of standard deviation and coefficient of variation [26].

*Data Processing*. The data collected was processed using Excel version 2013. Two mathematical models were tested to define the calibration curve: the quadratic function (*y* = *ax*^2^ + *bx* + *c*) and the logarithmic function (*y* = *a* ln(*x*) + *b*).

After the establishment of calibration models and the estimation of the coefficients, the inverse predicted concentrations were calculated, as well as the precision and trueness of data. Tolerance intervals were calculated for accuracy profile plotting.

The equations used to calculate the different validation parameters are presented in Supplementary Materials (available here). The methods of calculation of the fidelity and trueness criteria are presented in Table 4 [23].  Table 5 summarizes formulas used to calculate various components of the accuracy profile, including tolerance interval limits per level [23].

## 3. Results and Discussion

### 3.1. Method Optimization

#### 3.1.1. Mobile Phase Selection

During the mobile phase optimization, the dichloromethane-acetone-ammonium hydroxide system (20 : 1.5: 0.2, v/v/v) did not allow the elution of band-shaped deposits from the baseline after 30 min of plate development. With the second solvent system “dichloromethane-acetone-ammonium hydroxide” (10 : 10 : 0.2, v/v/v), the migration was less than 2 cm from the deposition line, with an *R*_*f*_ < 0.2. Therefore this mobile phase was not used in further developments. The use of chloroform-diethylamine- methanol system (15 : 3: 2, v/v/v), resulted in bands eluted to the solvent front.

**Figure 1:**
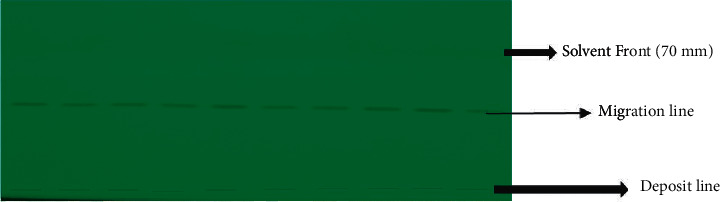
Chromatographic plate obtained with a mobile phase dichloromethane-methanol (9 : 1, v/v) and revealed at 254 nm.

The “dichloromethane and methanol” system in the ratio of 9 : 1 (v/v) gave the best migration profile with a frontal ratio between 0.5 and 0.6 (Figure 1).

This is in line with the optimal performance of the composition of a mobile phase that should allow a frontal ratio between 0.2 and 0.8 [24]. The presence of any trace between the deposition line and the eluted band indicates that the substance has been fully eluted (Figure 1). The frontal ratio average is 0.595 ± 0.015 for sildenafil under these mobile phase conditions. In a similar analysis of sildenafil by HPTLC, the authors found a frontal ratio of 0.53 ± 0.01 with a chloroform-methanol-diethylamine (90 : 10 : 1, v/v/v) mobile phase [27].

#### 3.1.2. Wavelength Selection

The areas under the curve obtained at the different wavelengths tested with solutions of different concentrations are shown in the supplementary material (Table 1, Supplementary material). The measurements on the same plate were repeated three times. At the wavelength of 305 nm, the areas of the peaks were the largest. This wavelength was chosen for further measurements of peak areas and peak heights. This wavelength seems the best for sildenafil analysis as other authors found the same in their work on the determination of sildenafil by HPTLC in aphrodisiac plant preparations [27].

#### 3.1.3. Assessment of Specificity

**Figure 2:**
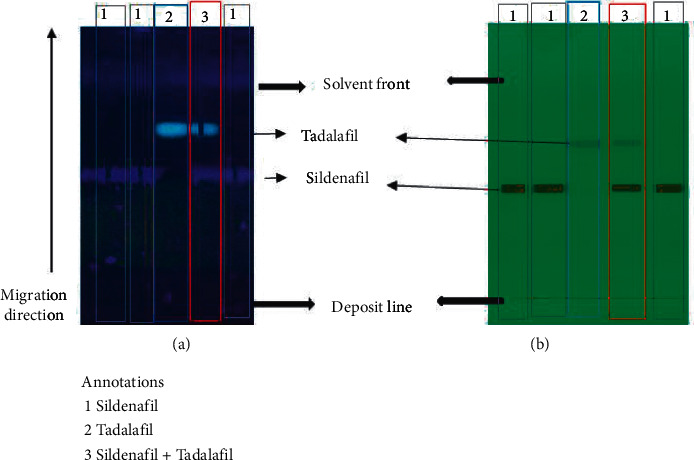
(a) Chromatographic plate revealed under 366 nm light. (b) Chromatographic plate revealed under 254 nm light.

**Figure 3:**
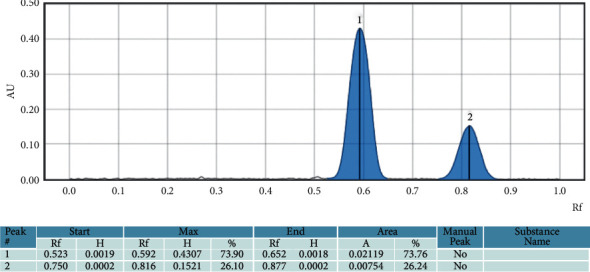
Chromatographic profile of the solution containing sildenafil and tadalafil.

Specificity was assessed by comparing the peak profile and the frontal ratio of sildenafil citrate with that of tadalafil, another phosphodiesterase type 5 inhibitor. The frontal ratios are respectively estimated at 0.592 ± 0.015 for sildenafil citrate and 0.806 ± 0.010 for tadalafil are shown in Figures 2 and 3. There is very good discrimination between the peaks of the two compounds and this was confirmed by the estimated resolution of 2.77 (Equation (1), Supplementary material).

Good separation of compounds observed on the chromatographic plates as well as the chromatogram allowed identifying unequivocally each of the two substances according to their frontal ratios. The method was then specific in determining the mixture composition.

The chromatograms obtained with the deposition of sildenafil, tadalafil, and the solution containing both substances showed the Gaussian profile for all peaks. The frontal ratio of Sildenafil was 0.589. The mean *R*_*f*_ of sildenafil was 0.592 ± 0.015. This is in the range of 0.2 and 0.8 as required for HPTLC separation. The frontal ratio of tadalafil is 0.815 with a mean of 0.806 ± 0.010.

### 3.2. Validation by Accuracy Profile

#### 3.2.1. Calibration Plan

The equations obtained for the response functions (calibration data) were made with the heights and the areas of the peaks on each of the five series. The mathematical models used and the equations of the calibration lines are presented in the supplementary material (Table 2, Supplementary material). The coefficients of determination (*R*^2^), close to 1, allow saying that the variability of the heights and areas of the peaks can be explained by these mathematical models.

#### 3.2.2. Linearity Profiles

The calculation of recovery lines is presented in Table 6. It was obtained using logarithmic and polynomial models.

The overlap lines related to equations (3) and (4) are those that best satisfy the linearity criteria according to ISO [28]. The slopes and coefficients of determination of these lines are close to 1 and their intercept is close to zero (0).

Therefore the logarithm function applied to the peak heights or the polynomial function applied to the areas could be used for the validation of the method. However, the linearity profile does not give guarantees that each future result will be close enough to the true value and that a systematic error could compensate a random error and vice versa.

#### 3.2.3. Construction of the Accuracy Profile

**Figure 4:**
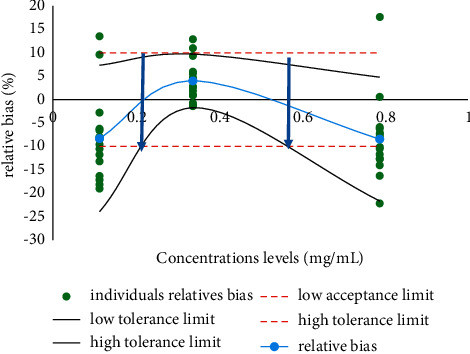
Accuracy profile obtained with the polynomial function applied to peak areas.

The accuracy profile was constructed based on the fidelity and trueness data. These data were calculated using logarithmic and polynomial functions based on peak areas and peak heights. The best fit is the one built with the polynomial function applied to the peak areas (Figure 4).

**Figure 5:**
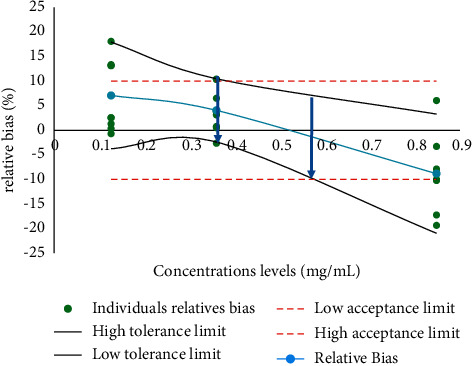
Accuracy profile obtained with the validation sample.

The range of concentrations obtained is between 0.2 and 0.6 mg/mL with the tolerance limits included in the acceptance limits. When using the logarithmic function and the polynomial function applied to peak heights, the working concentration ranges lead to results that are outside the tolerance and acceptance limits and therefore cannot be used for further validation. Therefore, the quadratic function was selected as the mathematical calibration model. For the validation of the method in the matrix, the Viagra® sample was a reference matrix, and the polynomial function applied to the peak areas was used. It allowed the construction of an accuracy profile establishing the validity interval between 0.4 and 0.6 mg/mL (Figure 5). The performance metrics for fidelity and accuracy are summarized in Tables 7 and 8. The limit of detection and limit of quantification were estimated to be 0.1157 mg/mL and 0.3821 mg/mL, respectively.

#### 3.2.4. Application of the Method to Samples

Collected samples were prepared at a concentration ±0.5 mg/mL. All samples analyzed contained sildenafil. Percentages of recovery are calculated and presented in Table 9. The method used allows not only identifying sildenafil in a product but also estimating the amount of sildenafil. Drug legal supply chains were all compliant in terms of drug content. These results may suggest that legal supply chains are secure. In the illicit market, both compliant and noncompliant drugs were found. Among the six (6) drug formulations from the illicit market analyzed, four (4) were noncompliant with three (3) underdosed and one (1) overdosed. Several reasons can explain these results, and more investigations are needed.

Quantitative analyses of most of the drugs from the illicit supply chain were above the maximum recommended dose in regard to efficacy and safety, and their use could cause several cardiovascular risks [29].

In addition to the satisfactory performance obtained (accuracy, recovery, and precision data shown above), the advantage of the present method over other reference chromatographic methods (especially HPLC) is the lower investment cost due to the relatively small volume of mobile phase used to analyze several samples at the same time on the same chromatographic plate. This also allows obtaining results very quickly.

## 4. Conclusion

The results obtained showed that the developed HPTLC method was specific and accurate for the determination of sildenafil. It could be used therefore under the conditions described in this study to identify and quantify active substances in counterfeit drugs. This study also reveals that illicit drug markets should be of concern for regulatory authorities as they represent a life threat for the population.

## Figures and Tables

**Table 1:** Characteristics of analyzed samples.

Drug supply circuit	Name, dosage, form	Regulatory status	Batch number	Expiry date
Legal	Plaevz 50 mg tablets B/4	Lack of marketing authorization	TET-1703	04/2020
	Cupid 50 mg tablets B/1	Valid marketing authorization	ET389E8002	12/2020
	Asmoforce 50 mg tablets B/4	Valid marketing authorization	MDTJL1701	05/2020
	Erector 50 mg tablets B/1	Valid marketing authorization	SIL05034	04/2020
	Erekta 50 mg tablets B/4	Valid marketing authorization	7218762	04/2020

Illegal	Venegra 50 mg tablets B/5	None		
	Yoga 100 mg tablets B/4			
	Meinnah 120 mg tablets B/5			
	Kdgra 120 mg tablets B/5			
	Rygra 120 mg tablets B/5			

**Table 2:** Optimized operating conditions for the determination of sildenafil.

Parameters	Value/selected characteristic(s)
Mobile phase	Methylene chloride-methanol (9 : 1, v/v)
Solubilization and dilution solvent	Methanol
Plates	Merck TLC silica gel F_254_ 20 × 10 cm
Deposited volume on the plate	5 *μ*L
Width of the deposit strip	7.4 mm
Length between deposits	9.4 mm
Distance from the deposition line to the edges of the plate (abscissa and ordinate)	8 and 10 mm
Development distance on plate	70 mm
Development mode	Linear ascending mode
Saturation time of mobile phase	30 min
Deposit flow	15 *µ*L/s
Deposit mode	Spray
Gas for sample application	Nitrogen
Detection wavelength	305 nm

**Table 3:** Validation plan.

Parameters	Number of analysis
Calibration plan (response function)	Five (5) series realized in the conditions of intermediate fidelity
	Four (4) levels of concentration
	Three (3) repetitions per concentration level
Validation plan (standard)	Five (5) series carried out under the conditions of intermediate fidelity
	Four (4) levels of concentration
	Three (3) repetitions per concentration level
Validation plan (sample)	Three (3) series carried out under the conditions of intermediate fidelity
	Four (4) levels of concentration
	Three (3) repetitions per concentration level

**Table 4:** Mode of calculation of the criteria of fidelity and trueness by level.

Criteria	Calculation methods	Levels		
		1	…	k
Intermediate fidelity standard deviation	*S*_*kFI*_=$\sqrt{\left(S_{kr+}^{2}S_{kB}^{2}\right)}$			
Intermediate fidelity variation coefficient	$\left(S_{kFI}/{\bar{\bar{x}}}_{k}\right)\times 100$			
Absolute mean bias	${\bar{\bar{z}}}_{k}-{\bar{\bar{x}}}_{k}$			
Relative average bias	$\left[{\bar{\bar{z}}}_{k}-{\bar{\bar{x}}}_{k}/{\bar{\bar{x}}}_{k}\right]$ × 100			
Average recovery rate	$\left({\bar{\bar{z}}}_{k}/{\bar{\bar{x}}}_{k}\right)\times 100$			

${\bar{\bar{z}}}_{k}$ : mean concentration found; ${\bar{\bar{x}}}_{k}$: mean reference value; *s*_*kr*_ : repeatability standard deviation; *s*_*kB*_ : interseries standard deviation.

**Table 5:** Modes of calculation of accuracy profile parameters.

Criteria	Calculation methods	Levels		
		1	…	k
Low tolerance limit	$\bar{\bar{z}}-K_{\text{tol}}\times s_{\text{IT}}$			
High tolerance limit	$\bar{\bar{z}}+K_{\text{tol}}\times s_{\text{IT}}$			
Relative low tolerance limit	$\left[\bar{\bar{z}}-k_{\text{tol}}\times s_{\text{IT}}/\bar{\bar{x}}\right]\times 100$			
Relative high tolerance limit	$\left[\bar{\bar{z}}+k_{\text{tol}}\times s_{\text{IT}}/\bar{\bar{x}}\right]\times 100$			
Low acceptability limit	(−*λ*)			
High acceptability limit	(+*λ*)			

*s*_IT_: standard deviation of the tolerance interval; *k*_tol_: tolerance interval coverage factor.

**Table 6:** Equations of the overlap lines.

	Logarithmic model		Polynomial model	
	Peak areas	Peak heights	Peak areas	Peak heights
Model equation	*y* = 1,356*x* − 0,0998	*y* = 1,033*x* + 0,0063	*y* = 0,9761*x* − 0,0028	*y* = 0,9183*x* + 0,0213
	*R* ^2^ = 0,9856	*R* ^2^ = 0,9985	*R* ^2^ = 0,9968	*R* ^2^ = 0,9836
	Equation (2)	Equation (3)	Equation (4)	Equation 5

**Table 7:** Precision data obtained with the polynomial function and the areas.

Concentration levels (mg/mL)	0.1125	0.3375	0.7875
Average found	0.1031	0.3509	0.7213
Repeatability standard deviation	0.004779	0.01253	0.06163
Intermediate fidelity standard deviation	0.01121	0.01508	0.07148
Repeatability coefficient of variation	4.632	3.571	8.544
Intermediate fidelity coefficient of variation	10.87	4.296	9.909

**Table 8:** Accuracy data obtained with the polynomial function and the areas.

Concentration levels (mg/mL)	0.1125	0.3375	0.7875
Average found by level	0.1031	0.3509	0.7213
Recovery	91.71%	103.99%	91.57%
High tolerance limit	49.40	9.911	5.2246
Low tolerance limit	−65.98	−1.931	−22.08
High limit of acceptability	10	10	10
Low limit of acceptability	−10	−10	−10

**Table 9:** Determination of sildenafil content in samples.

Channel type	Drug name and dosage	Recovery (%)			Compliance with Ph. Int. Specification (90–110%)
		Assay 1	Assay 2	Mean	
Legal distribution chain	Asmoforce 50 mg	91.25	96.81	94.03	Compliant
	Erekta 50 mg	94.82	95.31	95.06	Compliant
	Plaevz 50 mg	89.34	93.69	91.51	Compliant
	Cupid 50 mg	96.26	93.22	94.74	Compliant
	Erector 50 mg	105.9	107.9	106.9	Compliant

Illegal distribution chain	Meinnah 120 mg	69.15	70.11	69.63	Noncompliant
	Yoga 100 mg	95.94	93.56	94.75	Compliant
	Kdgra 120 mg	66.93	67.55	67.24	Noncompliant
	Rygra 120 mg	86.81	102.8	94.80	Compliant
	Venegra 50 mg	112.6	112.9	112.7	Noncompliant
	Kdgra 150 mg	86.61	84.35	85.48	Noncompliant

## Data Availability

The (mentioned or referenced) data used to support the findings of this study are included within the article and the supplementary information file.

## Supplementary Materials

Supplementary Materials Supplementary Materials. The additional data refer to two Tables (1 and 2) and the equations used for the calculation of the validation parameters of the method. In Table 1 (Supplementary material), the peak areas at different wavelengths and for three concentration levels are presented. The data show that the best linearity profile was recorded at 305 nm. In Table 2 (Supplementary material), the equations of the tested mathematical models are presented and the best results were obtained with the polynomial model applied to the peak areas.
