# Supplementary material for: Contribution to the Detection of Poor Quality Sildenafil Drugs in Burkina Faso Using High-Performance Thin-Layer Chromatography
Source: J Anal Methods Chem. 2021 Oct 13;2021:4093859. doi: 10.1155/2021/4093859 (PMC8528628; doi:10.1155/2021/4093859)
Supplement: Supplementary Materials — The additional data refer to two Tables (1 and 2) and the equations used for the calculation of the validation parameters of the method. In Table 1 (Supplementary material), the peak areas at different wavelengths and for three concentration levels are presented. The data show that the best linearity profile was recorded at 305 nm. In Table 2 (Supplementary material), the equations of the tested mathematical models are presented and the best results were obtained with the polynomial model applied to the peak areas. [file 4093859.f1.docx]

**Equations used to calculate the different validation parameters**

- Inverse concentration predicted with the quadratic function for y = ax^2^+bx+c

$$x=\frac{-b\pm\sqrt{b^{2}-4ac}}{2a}$$

- Inverse concentration predicted with the logarithm function where y = a ln(x)+ b

X = $e^{\left[ \frac{y-b}{a} \right]}$

- Resolution between two peaks: $Rs=\frac{x_{2}-x_{1}}{1/2(w_{1}+w_{2})}$ (equation 1) with x_1_ et x_2_ the heights of peaks 1 and 2, and w_1_ and w_2_ their widths at the base.
- Mean : 𝑥̅ =$\frac{\sum_{i}^{n} xi}{n}$ , with 𝑥̅ : arithmetic mean of a series of measurements, n : number of measurements;
- Standard deviation : S(*n)* = $\sqrt{\frac{\sum_{i}^{n} {(x̅ -xi)}^{2}}{n}}$ with 𝑥𝑖 : individual measures, S : Standard deviation of the measurement series

**Supplementary material, Table 1.** Peak areas at different wavelengths

| Parameters | Wavelength (nm) | | | | | |
| --- | --- | --- | --- | --- | --- | --- |
|  | 254 | 278 | 292 | 305 | 312 | 333 |
| Area (0.06 mg/mL) | 0.00202 | 0.00258 | 0.00410 | 0.00479 | 0.00453 | 0.00265 |
| CV(%) | 4.33.10^-8^ | 6.3.10^-7^ | 2.33.10^-8^ | 1.2.10^-7^ | 1.9.10^-7^ | 3.03.10^-7^ |
| Area (0.01 mg/mL) | 0.00048 | 0.00061 | 0.00100 | 0.00119 | 0.00110 | 0.00058 |
| CV(%) | 5.23.10^-7^ | 2.8.10^-7^ | 4.93.10^-7^ | 3.6.10^-7^ | 3.43.10^-7^ | 7.10^-8^ |
| Area (0.005 mg/mL) | 0.00031 | 0.00037 | 0.00058 | 0.00071 | 0.00063 | 0.00038 |
| CV(%) | 2.33.10^-8^ | 2.63.10^-7^ | 4.23.10^-7^ | 2.1.10^-7^ | 5.73.10^-7^ | 4.23.10^-7^ |

**Supplementary material,** **Table 2.** Mathematical models used and equations of the calibration lines

|  | Logarithmic model | | Polynomial model | |
| --- | --- | --- | --- | --- |
| Series | Peak areas | Peak heights | Peak areas | Peak heights |
| 1 | Y= 0.0116ln(x) + 0.035  R² = 0.9895 | Y= 0.1753ln(x) + 0.6482  R² = 0.9983 | Y= -0.0205x^2^ + 0.0522x + 0.0052  R² = 0.9997 | Y = -0.4397 x^2^ + 0.9235x + 0.1769  R² = 0.9904 |
| 2 | Y= 0.0113ln(x) + 0.0339  R² = 0.9916 | Y = 0.1751ln(x) + 0.6296  R² = 0.9993 | Y= -0.0233 x^2^ + 0.0541x + 0.0045  R² = 1 | Y= -0.419 x^2^ + 0.8983x + 0.1629  R² = 0.9897 |
| 3 | Y=0.01ln(x) + 0.031  R² = 0.9941 | Y= 0.1691ln(x) + 0.6657  R² = 0.9985 | Y= -0.0176 x^2^ + 0.0439x + 0.0055  R² = 0.9972 | Y= -0.5551 x^2^+ 1.0088x + 0.1977  R² = 0.9947 |
| 4 | Y=0.0123ln(x) + 0.0359  R² = 0.9855 | Y = 0.1757ln(x) + 0.6283  R² = 0.9986 | Y=-0.0203 x^2^ + 0.0533x + 0.0047  R² = 1 | Y= -0.4399 x^2^ + 0.9386x + 0.1546  R² = 0.9968 |
| 5 | Y=0.0093ln(x) + 0.0297  R² = 0.997 | Y= 0.1701ln(x) + 0.6529  R² = 0.9988 | Y= -0.0181 x^2^ + 0.0428x + 0.0057  R² = 0.9961 | Y= -0.4182 x^2^ + 0.8857x + 0.1975  R² = 0.9879 |
